# Quantifying the Viscosity of Individual Submicrometer Semisolid Particles Using Atomic Force Microscopy

**DOI:** 10.1021/acs.analchem.3c01835

**Published:** 2023-09-23

**Authors:** Chamika K. Madawala, Hansol D. Lee, Chathuri P. Kaluarachchi, Alexei V. Tivanski

**Affiliations:** Department of Chemistry, University of Iowa, Iowa City, Iowa 52242, United States

## Abstract

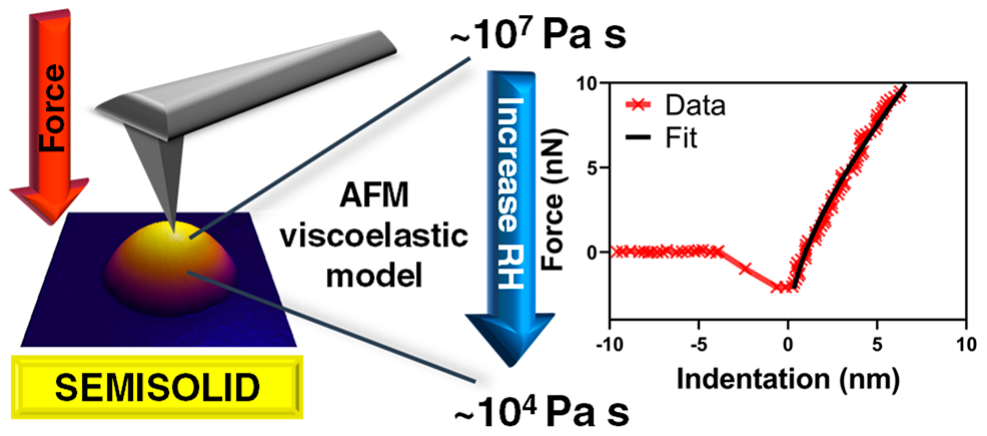

Atmospheric aerosols’ viscosities can vary significantly depending on their composition, mixing states, relative humidity (RH) and temperature. The diffusion time scale of atmospheric gases into an aerosol is largely governed by its viscosity, which in turn influences heterogeneous chemistry and climate-relevant aerosol effects. Quantifying the viscosity of aerosols in the semisolid phase state is particularly important as they are prevalent in the atmosphere and have a wide range of viscosities. Currently, direct viscosity measurements of submicrometer individual atmospheric aerosols are limited, largely due to the inherent size limitations of existing experimental techniques. Herein, we present a method that utilizes atomic force microscopy (AFM) to directly quantify the viscosity of substrate-deposited individual submicrometer semisolid aerosol particles as a function of RH. The method is based on AFM force spectroscopy measurements coupled with the Kelvin–Voigt viscoelastic model. Using glucose, sucrose, and raffinose as model systems, we demonstrate the accuracy of the AFM method within the viscosity range of ∼10^4^–10^7^ Pa s. The method is applicable to individual particles with sizes ranging from tens of nanometers to several micrometers. Furthermore, the method does not require prior knowledge on the composition of studied particles. We anticipate future measurements utilizing the AFM method on atmospheric aerosols at various RH to aid in our understanding of the range of aerosols’ viscosities, the extent of particle-to-particle viscosity variability, and how these contribute to the particle diversity observable in the atmosphere.

Atmospheric aerosols play an important role in radiative forcing either directly by scattering, reflecting, or absorbing solar radiation or indirectly by acting as cloud condensation nuclei (CCN) or ice nucleating particles (INPs) to facilitate cloud formation.^1−6^ These climate-relevant aerosol effects depend on the physicochemical properties of individual aerosols, such as morphology, mixing state, size, composition, phase state and viscosity,^7−9^ all in turn can vary depending on the surrounding relative humidity (RH) and temperature.^10−12^ In particular, the phase state of aerosols (i.e., solid, semisolid, and liquid) is important as it can regulate reactivity with atmospheric gases and control their CCN and water uptake behavior, and their ability to act as INPs.^3−5,10,13−16^

Previously, Lee et al. developed a methodology that allows accurate determination of the phase state of substrate-deposited individual submicrometer aerosol particles as a function of RH using atomic force microscopy (AFM).^3^ However, despite the ability to accurately determine the aerosol phase state, within a particular phase state, there is a wide range of viscosities. Specifically, solid aerosols have viscosity values greater than 10^12^ Pa s, semisolid aerosols have viscosity ranging between 10^12^ Pa s and 10^2^ Pa s, while liquid aerosols have viscosities between 10^2^ Pa s and 10^–3^ Pa s.^17^ Aerosol viscosity determines the equilibrium time scale at which atmospheric gas molecules diffuse into and out of aerosols, thus influencing the rate and type of heterogeneous reactions (e.g., surface or bulk oxidation), and subsequently their ability to act as efficient CCN or INPs.^10,17−19^ In that regard, quantification of the aerosol viscosity in semisolid state is particularly important because a large fraction of atmospheric aerosols are semisolid.^7,20^ Furthermore, given the wide viscosity range (10 orders of magnitude) of semisolid aerosols, the diffusion time scale can vary from seconds to years depending on a particular aerosol viscosity.^6,17^ Thus, semisolid aerosols with different viscosities could undergo different type and extent of atmospheric aging, which in turn would modify their cloud forming properties.^17,21^ Therefore, measurements of aerosol viscosity are needed, especially in the semisolid viscosity range. Such measurements need to be performed under varying RH to account for the variable nature of RH in the atmosphere. Furthermore, such measurements need to be performed on submicrometer sized aerosol particles, due to their significant lifetime in the atmosphere.^22^ The viscosity of submicrometer particles must be quantified directly without extrapolation from the measurements over supermicron counterparts, as aerosols often exhibit size-dependent composition, which alters their viscosity.^8^ Furthermore, viscosity measurements ideally need to be performed on a single particle basis as atmospheric aerosols from the same source and similar size range can exhibit particle-to-particle variability.^7,8,20,23^

Direct measurements of bulk liquid viscosity are typically done using viscometers, which are well established, inexpensive, and easy to use.^6,9,24^ However, the minimum volume of liquid required is far too large for atmospheric aerosols.^9^ The method is also largely constrained to quantifying viscosities below 10^8^ Pa s.^25^ There are several methods that can be used to measure the viscosity of individual aerosols. One such method is bead-mobility technique,^18,19,26^ where 30–50 μm individual particles are injected with 1 μm melamine beads. A gas flowing over the particle surface circulates the beads, and the bead velocity is an indicator of particle viscosity. This measurement can be performed to quantify viscosity within the range from 10^–3^ to 10^3^ Pa s. Second method is the poke-and-flow technique,^27−29^ where a supermicrometer particle is first indented with a needle and then the time required to reestablish the equilibrium shape is measured to quantify the viscosity within the range from 10^3^ to 10^7^ Pa s. However, a significant limitation for both methods is that it is applicable only to aerosols that are generally greater than tens of micrometers in size. Third method is based on the optical tweezers,^19,27,30−32^ where two supermicrometer aerosols are trapped and then coalesced to measure the relaxation time, which in turn can be used to quantify the aerosol viscosity within the range from 10^–3^ to 10^9^ Pa s. However, similar to the bead-mobility and poke-and-flow techniques, the optical tweezers method is not applicable for submicrometer aerosols. Finally, AFM was recently used to measure viscosity of aerosol droplets as a function of temperature by recording the resonant frequency response of AFM cantilever submerged into the droplet, which in turn enables quantification of viscosity of individual droplets.^33^ However, while the method is applicable to submicrometer aerosols, it is limited to the viscosity values below 10^–2^ Pa s. Collectively, no method currently exists that enables direct quantification of viscosity of individual submicrometer semisolid aerosols as a function of RH; the development of such new method is the focus of this study.

Recently, a linear three-dimensional Kelvin–Voigt viscoelastic model was developed, which relates pressures and mechanics under load to account both elastic and viscoelastic deformations within set boundary conditions.^28,29^ The application of Kelvin–Voigt model to AFM was previously developed toward quantitative determination of viscoelastic response of various systems such as biological cells, biofilms, and polymer blends.^28,29,34−36^ Specifically, Garcia et al. applied this model to quantify using AFM the Young’s modulus and viscosity of individual biological cells. In particular, they reported quantification of the Young’s modulus (1 kPa–6 kPa) and viscosity (60 Pa s–460 Pa s) of individual cells, where the results overlapped well with the finite element simulations, confirming applicability of the method to quantify viscosity of relatively soft systems.^28,29^ Herein, for the first time, the same viscoelastic theory was developed to quantify the viscosity of environmentally relevant, much stiffer individual submicrometer particles. Submicrometer model aerosols containing glucose, sucrose, and raffinose were chosen for this study for two reasons. First, these compounds are commonly present in secondary organic aerosols and sea spray aerosols.^37,38^ Second, previously published works have established an accurate relationship between the RH and corresponding viscosity for each of these systems, thus enabling us to directly compare AFM based viscosity quantification at various RH with the literature.^30^ For these model systems, AFM force measurements were performed over individual substrate-deposited submicrometer particles at various RH values to yield force plots, which then were used to quantify single particle viscosity at a particular RH at room temperature (20–25 °C) using Kelvin–Voigt viscoelastic model. The results indicate that the viscosity measurements using AFM are applicable for semisolid individual submicrometer aerosols within the viscosity range of ∼10^4^–10^7^ Pa s.

## Experimental Section

### Particle Generation

Sucrose, glucose, and raffinose were purchased from Sigma-Aldrich, as reagent grade 99.99% purity. All chemicals were used without additional purification, and they were dissolved in deionized water (with a resistivity of 18 MΩ·cm, indicating high water purity with minimal impurities) at a 1 mM molar concentration. Sucrose, glucose, and raffinose aerosols were generated with a constant output atomizer (TSI, Model 3076) from 1 mM aqueous solutions. The aerosol stream was mixed with wet air at a constant rate of 20 L/min to achieve ∼80% RH in the mixing chamber and then aerosols were deposited by impaction using a Micro Orifice Uniform Deposit Impactor (MOUDI; MSP, Inc., Model 110).^3,4,8,39^ Particles were deposited on hydrophobically coated (Rain-X) silicon wafers (Ted Pella Inc., part no. 16008) placed on the MOUDI stage 7, which corresponds to an aerodynamic diameter range of 0.3–0.56 μm. The substrate-deposited particles were stored in clean Petri dishes and kept inside a laminar flow hood (NuAire, Inc., NU-425-400) at room temperature (20–25 °C) and ambient pressure at 20–25% RH, and AFM experiments were performed on the same or following day.^40^

### Atomic Force Microscopy Imaging and Force Measurements

A molecular force probe 3D AFM (Asylum Research, Santa Barbara, CA) was used for imaging and force measurements at ambient temperature (20–25 °C) and varying RH ranging from ∼10% to 60% using a custom-made humidity cell.^41^ Silicon nitride AFM probes (MikroMasch, Model CSC37) with a nominal spring constant of 0.35 N/m and a tip radius of curvature of 10 nm were used for both imaging and force measurements. Actual spring constants were quantified using the thermal noise method.^42^ All samples were first imaged in the AC (tapping) mode to locate individual particles and quantify their size at ∼10% RH. AFM force measurements were next performed in contact mode by measuring forces acting on the AFM tip as a function of vertical piezo displacement (i.e., force plots) as the tip moved toward and away from an approximate center of a particle (see Figure S1 for more details). Scan rate was 1 Hz for all of the force measurements. For force measurements, at least 5 repeated force-vertical piezo displacement curves with a typical maximum applied loading force of 20 nN were collected at each selected RH ranging from ∼10% to 60%. The equilibration time after each change in RH was approximately 10–15 min to ensure the particles are in thermodynamic equilibrium with surrounding water vapor at a particular RH.^3,4,41^ For each sample at a particular RH, the approach data in the contact region, where the tip is indenting into the particle, were used to determine the particle viscosity at the corresponding RH. The viscosity values were determined for each saccharide system at a particular RH, with each value reported as an average and two standard deviations. The RH-viscosity relationships for glucose, sucrose and raffinose were taken from Song et al.^30^ and used for comparison with the AFM-based measurements.

## Results and Discussion

### Atomic Force Microscopy Viscoelastic Model for Viscosity Measurements Based on the Kelvin–Voigt Viscoelastic Model

The linear three-dimensional Kelvin–Voigt model was previously developed to successfully quantify the viscoelastic response of various relatively soft systems including biofilms, polymer blends and biological cells using AFM.^28,29,34^ The theory relates pressures and mechanics under load to account for the elastic and viscoelastic deformations of a sample within set boundary conditions. The general analytical expression for the linear three-dimensional Kelvin–Voigt viscoelastic model is following:^34^
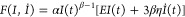
1where *F*(*I*, *İ*) is the force at a particular indentation distance (*I*) at time *t*, *İ* is the first derivative of indentation distance with respect to time (i.e., rate of indentation), α and β are the tip-geometry and sample thickness related coefficients, respectively, and *E* and η is the Young’s modulus and viscosity of the sample, respectively.^28^ Here, we aim to extend this method toward quantification of viscosity as a function of RH for much stiffer (Young’s moduli several orders of magnitude higher than that for biological systems) atmospherically relevant individual submicrometer aerosol particles.^13^ Using the linear three-dimensional Kelvin–Voigt model and assuming AFM tip geometry as a sphere of radius *R* (tip radius of curvature) and using the Hertzian elastic contact mechanics model that accounts for the surface adhesion contribution, the following expression can be derived:^28,34,43,44^
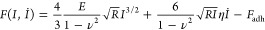
2where ν is the particle Poisson’s ratio and *F*_adh_ is the adhesion force between the tip and particle. Finally, by adding varying RH as an additional experimentally controlled variable that is assumed to modify the force, indentation distance, rate of indentation, Young’s modulus, viscosity, and adhesion force, one obtains:
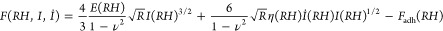
3Then, at a particular RH, AFM force plots data obtained over an individual particle can be utilized to simultaneously measure force as a function of indentation distance (Figure S1), indentation distance as a function of time, and adhesion force between the AFM tip and particle. The variables *I*, *İ*, *E*, η, and *F*_adh_ are considered to be functions of RH. The Poisson’s ratio of each model saccharide system studied here was assumed to be RH-independent and equal to 0.3.^13,45^ At a particular RH, if the indentation distance versus time plot is linear (as will be shown below), then the data can be fit to a straight line, yielding the slope equal to the rate of indentation. Finally, force versus indentation distance data can be fit to eq 3, yielding the particle viscosity and Young’s modulus at corresponding RH. Noteworthy, since the model assumes AFM tip as a sphere with the radius corresponding to tip radius of curvature, the maximum indentation distance for the analysis should be limited to the tip radius of curvature. Next, the applicability of the model to accurately quantify viscosity is tested by performing AFM measurements on individual submicrometer particles of glucose, sucrose, and raffinose as a function of RH.

### Quantification and Validation of Viscosity as a Function of RH for Individual Submicrometer Glucose, Sucrose, and Raffinose Particles

**Figure 1:**
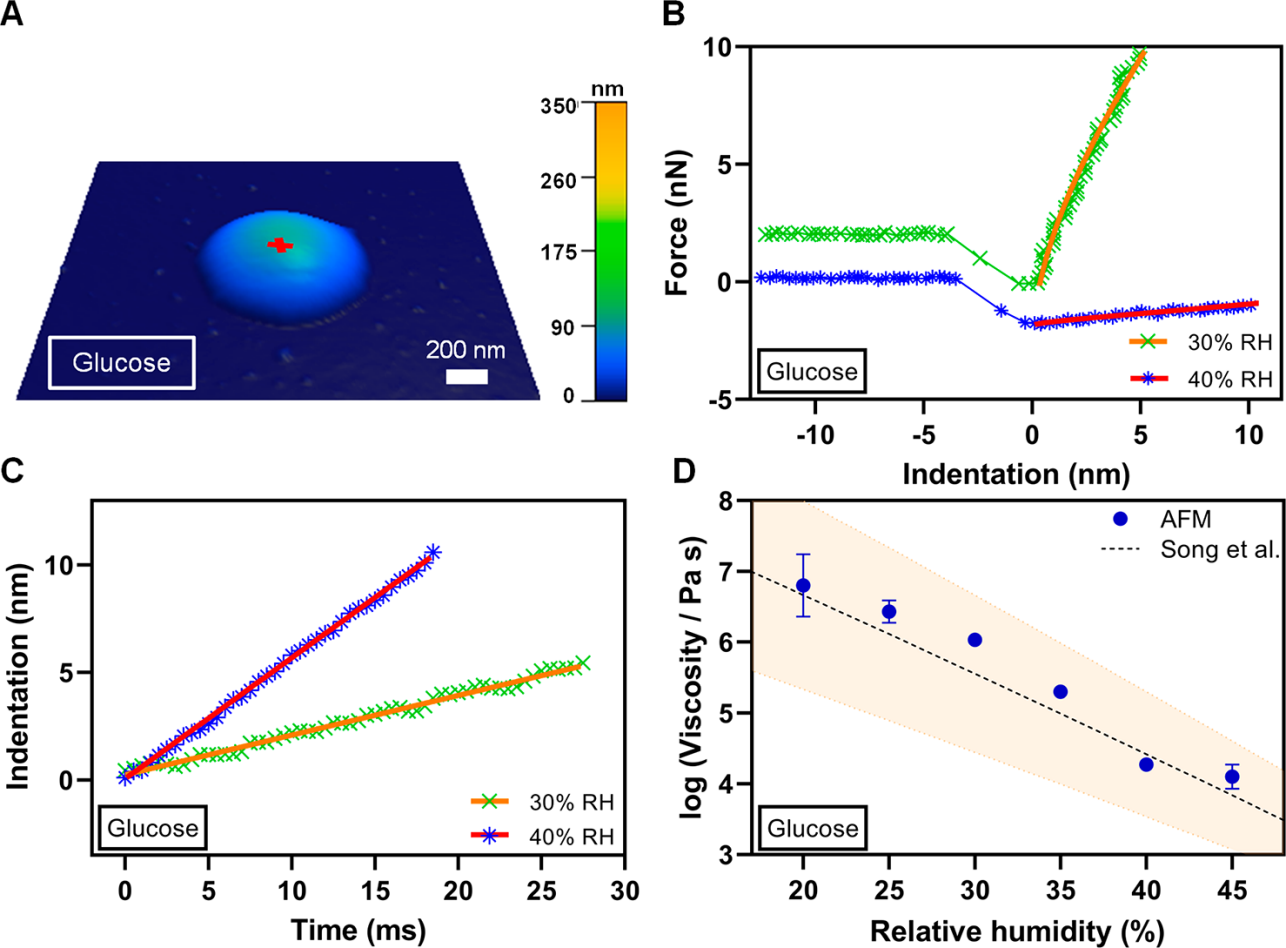
(A) Representative AFM 3D height image at ∼10% RH of a single glucose particle displaying rounded morphology with the height of ca. 100 nm and base size of 1100 nm. The red cross corresponds to an approximate center of the particle where force measurements were performed. (B) Measured force versus indentation distance data (symbols) along with the fit line using eq 3 at 30% RH (green crosses and orange line) and 40% RH (blue asterisks and red line). The force data at 30% RH were offset by 2 nN for clarity. Only approach to the particle surface data is shown. (C) Measured indentation distance versus time (symbols) along with the linear fit line at 30% RH (green crosses and orange line) and 40% RH (blue asterisks and red line) used to quantify the first derivative of indentation distance with respect to time. (D) Viscosity quantified from the AFM force measurements (blue circles are average values and error bars are two standard deviations) as a function of RH at room temperature (20–25 °C) along with the expected values from Song et al.^30^ (black dashed line), Copyright 2016 American Chemical Society, where orange shadow area represents ±10% uncertainty. For some viscosity data points, the standard deviation is smaller than the size of the symbol.

Figure 1A shows a representative AFM 3D height image of an individual glucose particle at ∼10% RH displaying a rounded morphology with a height of ca. 100 nm and base size of ca. 1100 nm. Figure 1B shows representative force versus indentation distance plots (symbols are data) as tip approaches to the particle surface (i.e., approach data) measured at 30% and 40% RH over an approximate center of the glucose particle shown in Figure 1A. The force data at 30% RH are offset by 2 nN for clarity. The zero-indentation distance that corresponds to the point of contact between the AFM tip and the particle surface was determined using the Hertzian elastic contact model (Figure S1). Force data collected at positive indentation distances correspond to the contact region between the tip and particle surface and were used for the viscosity quantification. Figure 1C shows the indentation distance at the contact region versus time at 30% and 40% RH (symbols are data) collected simultaneously with the force versus indentation distance data shown in Figure 1B. The zero time corresponds to zero indentation distance when the AFM tip just contacts the particle surface. Each indentation distance versus time plot is clearly linear and thus was fit to a straight line (solid lines are fit), yielding the slope which is equal to *İ* (rate of indentation) of 220 ± 2 nm/s (*R*^2^ = 0.994) and 560 ± 3 nm/s (*R*^2^ = 0.999) for 30% and 40% RH, respectively. Next, using the determined *İ* values at these two RH, the force data at 30% and 40% RH shown in Figure 1B were fit using eq 3 (solid lines are fit), yielding the viscosity of 10^6.02±0.01^ Pa s and Young’s modulus of 31 ± 3 MPa at 30% RH (*R*^2^ = 0.995), and the viscosity of 10^4.3±0.1^ Pa s and Young’s modulus of 2.6 ± 1.1 MPa at 40% RH (*R*^2^ = 0.982). Here, the uncertainty for each value is based on the fit of a single force profile. An increase in *İ* and decrease of viscosity and Young’s modulus values with increasing RH is consistent with water uptake, which decreases solute concentration, makes particle softer (lower Young’s modulus) and less viscous.^46^ The data analysis was then repeated for a total of three repeated force–indentation measurements at each RH, yielding the average and two standard deviations for the viscosity as 10^6.03±0.01^ Pa s and 10^4.27±0.08^ Pa s, and Young’s modulus as 23 ± 4 MPa and 2 ± 0.3 MPa for at 30% and 40% RH, respectively. The AFM determined averaged viscosity values at these RH can be compared with previously reported results from Song et al.^30^ Specifically, the viscosity values from Song et al. were 10^5.6±1.1^ and 10^4.4±0.9^ Pa s at 30% and 40% RH, respectively, which is in excellent agreement with the AFM determined viscosity results at these RH, thus confirming the applicability of the method.

To further assess the applicability and accuracy of the AFM method, force–indentation measurements and data analysis identical to those described above for 30% and 40% RH were performed on the same glucose particle over a wider range of RH from ∼10% to 55%. The following criteria were established to identify the suitability of a particular force plot for the viscosity determination using the AFM viscoelastic model. First, at lower RH, a limited water uptake is expected, thus the particle response to tip indentation is largely governed by the elastic contribution term (i.e., first term in eq 3). On the other hand, at elevated RH, the particle becomes progressively more hydrated, ultimately forming a liquid droplet, and the particle response to tip indentation is largely governed by the viscosity contribution term (i.e., second term in eq 3). Since applicability of the model depends on nonnegligible contribution of both elastic and viscosity terms, it is limited to particles in the semisolid phase state where both terms are relevant. Previously, AFM was used to successfully identify phase states of substrate deposited particles.^3,9^ Quantitative analysis of phase state was established by measuring RH-dependent viscoelastic response distance (VRD) and relative indentation depth (RID) from AFM force profiles collected over an individual particle.^3^ The VRD is defined as the difference in the tip–sample separation recorded at 0 nN force between the approach and retraction force data. The RID is defined as the ratio of indentation distance at a specific force to corresponding maximum particle height recorded from AFM 3D height image.^3^ Based on the VRD and RID analysis and assuming non-negligible contributions from both the elastic and viscosity terms to the AFM viscoelastic model, the following was experimentally established: a particular force plot is suitable for the viscosity characterization if the corresponding VRD (at 20 nN) is greater than 1.4 nm and the RID (at 10 nN) is less than 0.93. These criteria establish a relatively quick and straightforward assessment to identify force profiles that are expected to be suitable toward viscosity quantification using AFM viscoelastic model. Thus, if these force profiles criteria are valid, we expect non-negligible contributions from each term in eq 3, and therefore, the viscoelastic model is expected to be applicable, as we demonstrate next.

Based on the above criteria using VRD and RID, the RH range applicable for the viscosity quantification for glucose was determined to be from 20% to 45%. For force plots collected at and below 15% RH, and at and above 50% RH, either VRD or RID values were outside of the established criteria range. Figure 1D shows how viscosity determined using AFM varies over 20%–45% RH range along with the corresponding viscosity values reported by Song et al.^30^ The AFM viscosity data are in excellent agreement with the expected values over this RH range; thus, we can conclude the AFM viscoelastic method is applicable to accurately quantify viscosity of individual glucose particles within the range of 10^4.1^–10^6.8^ Pa s over 20%–45% RH.

**Figure 2:**
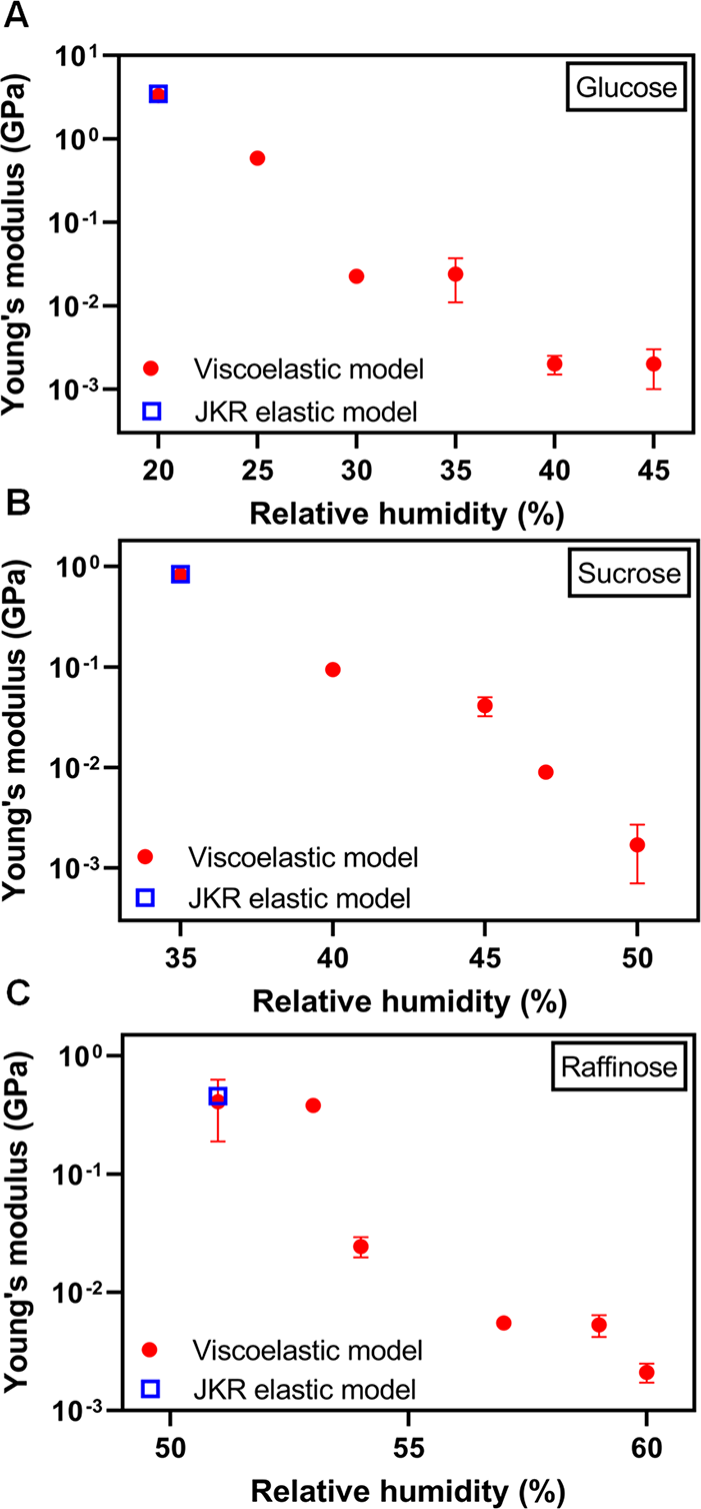
Young’s modulus quantified from the AFM viscoelastic model (red circles are average values and error bars are one standard deviation) as a function of RH at room temperature (20–25 °C) along with Young’s modulus values from the Johnson–Kendall–Roberts (JKR) elastic model (blue squares) for (A) glucose, (B) sucrose, and (C) raffinose model single particle systems. For some Young’s modulus data, the standard deviation is smaller than the size of the symbol. The comparison with the JKR elastic model is shown at the lowest RH value where viscoelastic model was applicable at 20%, 35%, and 51% for glucose, sucrose, and raffinose, respectively.

Figure 2A shows how the Young’s modulus of single particle glucose determined using the AFM viscoelastic model varies with respect to RH over 20%–45% RH range. The Young’s modulus values clearly decrease with increase in RH, from 3.3 ± 0.4 GPa at 20% RH to 2 ± 1 MPa 45% RH. As mentioned above, the decrease is expected due to increasing water uptake at higher RH, which leads to the reduction of glucose concentration and in turn makes the particle softer.^3^ To confirm the determined Young’s modulus values are reasonable, we utilized the Johnson–Kendall–Roberts (JKR) elastic contact model to fit the approach force plot data to determine Young’s modulus of glucose particle at 20% RH using a previously established method.^47,48^ The Young’s modulus determined with the JKR model at 20% RH was found to be 3.5 ± 0.5 GPa, which is reasonably close to the 3.3 GPa value determined using the AFM viscoelastic model. Thus, we collectively confirm that the AFM viscoelastic model yields both accurate viscosity measurements and Young’s modulus results that are consistent with expectations, confirming the applicability of both terms in eq 3.

**Figure 3:**
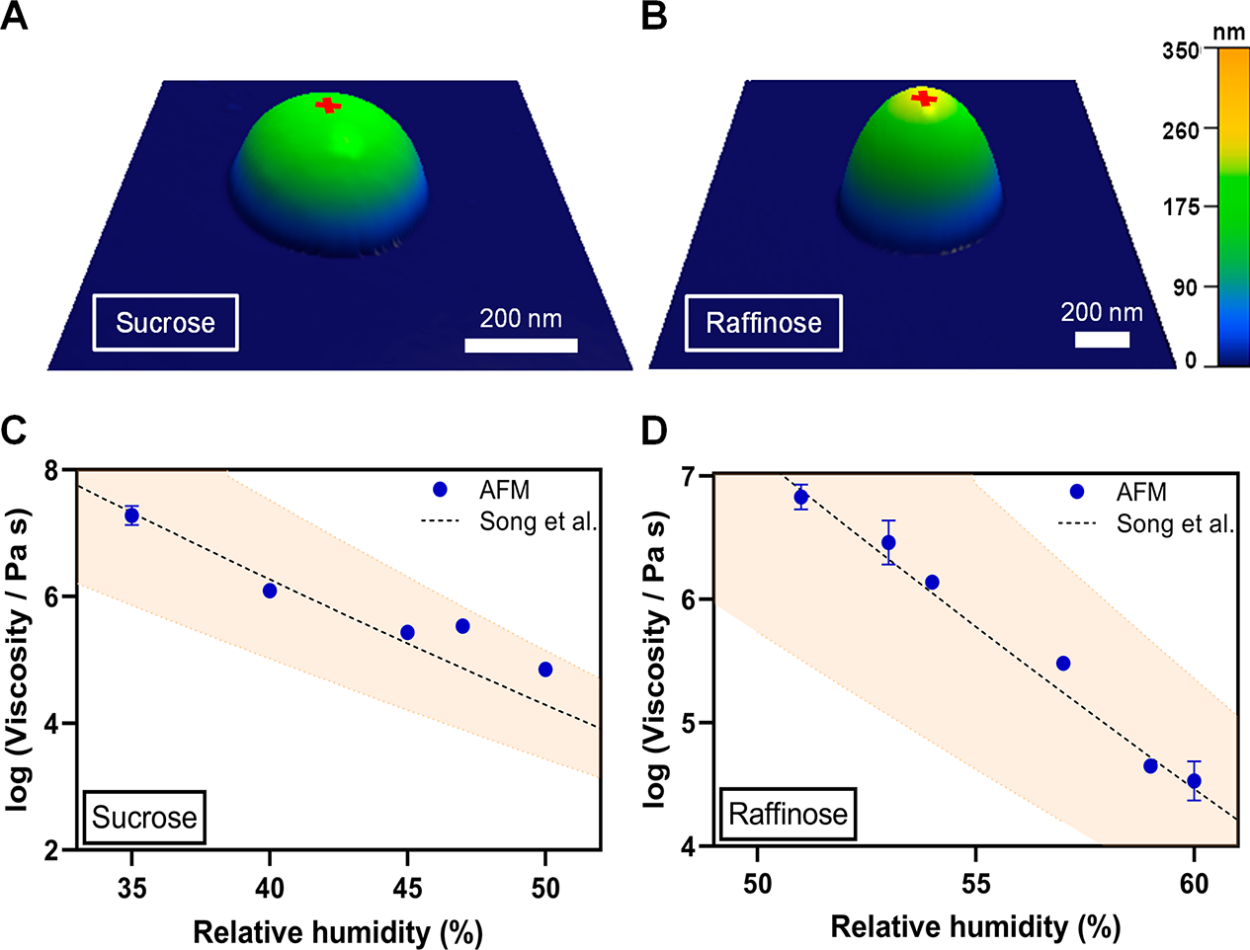
(A) Representative AFM 3D height images at ∼10% RH of sucrose particle with the height of ca. 200 nm diameter of 460 nm and (B) raffinose particle with the height of ca. 250 nm and diameter of 700 nm. The red cross corresponds to an approximate center of the particle where force measurements were performed. Viscosity quantified from force versus indentation distance data using AFM (blue circles are average values and error bars are two standard deviations) for (C) sucrose and (D) for raffinose as a function of RH at room temperature (20–25 °C) along with the expected values from Song et al.^30^ (black dashed line), Copyright 2016 American Chemical Society, where orange shadow area represents ±10% uncertainty. For some viscosity data points, the standard deviation is smaller than the size of the symbol.

To further validate the applicability of the AFM viscoelastic model, two additional model systems of single particle sucrose and raffinose were studied. Figure 3A, B shows representative AFM 3D height images of individual particles of sucrose (height of ca. 200 nm and diameter of ca. 460 nm) and raffinose (height of ca. 250 nm and diameter of ca. 700 nm) at ∼10% RH, respectively, each displaying rounded morphology. The rounded morphology for each system is consistent with previous studies.^13^ Based on the VRD and RID criteria for the selection of suitable force plots, the RH range applicable for the viscosity quantification for sucrose was determined to be from 35% to 50%. For sucrose force plots collected at and below 29% RH, and at and above 54% RH, either VRD or RID values were outside of the established criteria range. Similarly, the RH range applicable for raffinose was determined to be from 51% to 60%. For raffinose force plots collected at and below 47% RH, and at and above 63% RH, either VRD or RID values were outside the established criteria range.

Next, sucrose and raffinose force plots collected for the RH range of 35%–50% and 51%–60%, respectively, were analyzed using the approach described above to quantify the average viscosity and Young’s modulus at various RH values within these humidity ranges. Figure 3C, D shows how sucrose and raffinose viscosity determined using AFM varies within these humidity ranges along with the expected viscosity values from Song et al.^30^ For both saccharide systems, the AFM viscosity data are in excellent agreement with the expected values over these RH ranges. Thus, we can conclude the AFM viscoelastic method is applicable to accurately quantify viscosity of individual sucrose particles within the range of 10^4.9^–10^7.3^ Pa s over 35%–50% RH and viscosity of individual raffinose particles within the range of 10^4.5^–10^6.8^ Pa s over 51%–60% RH.

Figure 2B, C shows how the Young’s modulus of single particle sucrose and raffinose determined using the AFM viscoelastic model varies with respect to RH over 35%–50% and 51%–60% humidity range, respectively. The Young’s modulus decreases with an increase in RH for both model systems. Specifically for sucrose, Young’s modulus decreases from 0.82 ± 0.04 GPa at 35% RH to 2 ± 1 MPa 50% RH. For raffinose, Young’s modulus decreases from 0.41 ± 0.22 GPa at 51% RH to 2 ± 1 MPa 60% RH. As previously discussed, the decrease is expected due to increasing water uptake at higher RH, which leads to the reduction of concentration of sucrose or raffinose and in turn make particles softer.^3^ Similar to above, to confirm the determined Young’s modulus values are reasonable, we utilized the JKR elastic contact model to fit approach force plots data to determine Young’s modulus of sucrose and raffinose particles at 35% and 51% RH using previously established method.^47,48^ The Young’s modulus determined with the JKR model at 35% RH for sucrose is 0.84 ± 0.2 GPa and that for raffinose was found to be 0.46 ± 0.01 GPa at 51% RH. These values are reasonably close to Young’s moduli of 0.82 and 0.41 GPa determined using the AFM viscoelastic model for sucrose and raffinose, respectively.

Collectively, the results of the viscosity quantification based on glucose, sucrose, and raffinose single particle model systems indicate the AFM viscoelastic model is applicable to accurately quantify viscosity within the range of ∼10^4^–10^7^ Pa s, encompassing a significant portion of the semisolid phase state (10^2^–10^12^ Pa s). We previously established the AFM-based VRD and RID framework that allows us to determine the phase state of individual particles with the viscosity range of >10^12^ Pa s (solid), 10^12^–10^2^ Pa s (semisolid), and <10^2^ (liquid),^3^ and the viscoelastic model developed here further provides accurate viscosity quantification for semisolid particles ranging from ∼10^4^–10^7^ Pa s.

## Conclusions

In this work, the applicability and accuracy of the AFM force spectroscopy-based methodology in quantifying the viscosity of model submicrometer individual aerosol particles was explored. The method is based on first measuring forces as a function of indentation distance (force plots) as AFM tip indents into an individual particle of interest and then utilizing the Kelvin–Voigt viscoelastic theory to quantify the viscosity and Young’s modulus at a particular RH. Three model systems were studied: single particles of glucose, sucrose, and raffinose. We demonstrate that the AFM viscoelastic method can accurately measure viscosity of individual submicrometer semisolid particles as a function of RH within the viscosity range of ∼10^4^–10^7^ Pa s. Furthermore, we show that the AFM viscoelastic model can be utilized to quantify the elastic modulus of individual semisolid particles as a function of RH. Significantly, the method does not require prior knowledge of the composition of the particles. Collectively, the method is expected to facilitate viscosity measurements of various individual atmospheric aerosols and gain important insights into how the aerosol viscosity varies as a function of aerosol type, mixing states, size. Such studies can contribute to a better understanding of the role of viscosity of atmospheric aerosols and its impact on the climate and atmospheric processing.

## Data Availability

The data for this publication can be retrieved from the University of California, San Diego Library Digital Collection. https://doi.org/10.6075/J03T9HDN.

## Supporting Information Available

AFM force plots showing force versus vertical piezo displacement curve and force versus indentation curve collected over an individual glucose particle (PDF)
