# Supplementary material for: Quantifying the Viscosity of Individual Submicrometer Semisolid Particles Using Atomic Force Microscopy
Source: Anal Chem. 2023 Sep 23;95(39):14566–72. doi: 10.1021/acs.analchem.3c01835 (PMC10551855; doi:10.1021/acs.analchem.3c01835)
Supplement: Supplementary file 1 — ac3c01835_si_001.pdf [file ac3c01835_si_001.pdf]

QUANTIFYING THE VISCOSITY OF INDIVIDUAL SUBMICROMETER SEMISOLID PARTICLES USING ATOMIC FORCE MICROSCOPY

*Chamika K. Madawala, Hansol D. Lee, Chathuri P. Kaluarachchi, Alexei V. Tivanski**

Department of Chemistry, University of Iowa, Iowa City, Iowa 52242, USA.

Corresponding Author

*E-mail: alexei-tivanski@uiowa.edu

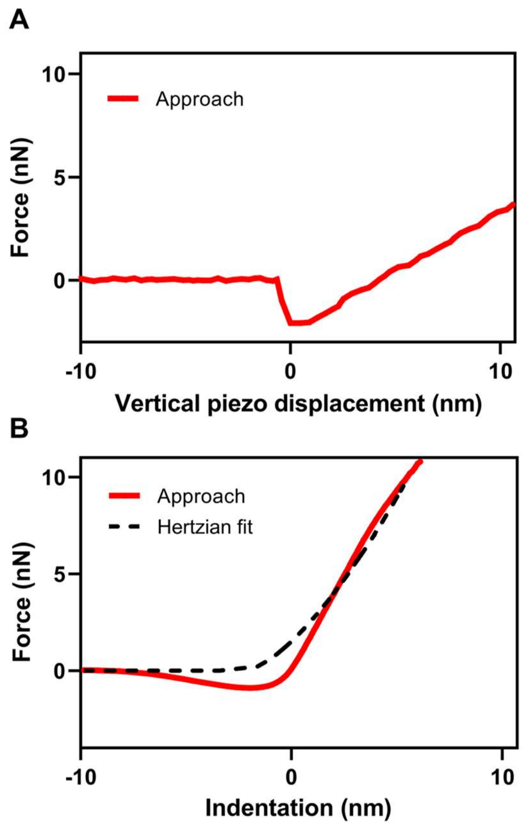

Figure S1. (A) Representative force versus vertical piezo displacement and (B) corresponding force versus indentation profiles (only approach data are shown) collected at 30% RH over an individual glucose particle. The black dashed line is the Hertzian elastic contact model fit used to determine point of contact (i.e., zero indentation).
